# Reduced gluteofemoral (subcutaneous) fat mass in young Japanese women with family history of type 2 diabetes: an exploratory analysis

**DOI:** 10.1038/s41598-022-16890-0

**Published:** 2022-07-22

**Authors:** Mari Honda, Ayaka Tsuboi, Satomi Minato-Inokawa, Mika Takeuchi, Miki Kurata, Bin Wu, Tsutomu Kazumi, Keisuke Fukuo

**Affiliations:** 1 Open Research Center for Studying of Lifestyle-Related Diseases, Mukogawa Women’s University, Nishinomiya, Hyogo Japan; 2 Department of Health, Sports, and Nutrition, Faculty of Health and Welfare, Kobe Women’s University, Kobe, Hyogo Japan; 3 Research Institute for Nutrition Sciences, Mukogawa Women’s University, 6-46, Ikebiraki-cho, Nishinomiya, Hyogo 663-8558 Japan; 4 Department of Nutrition, Osaka City Juso Hospital, Osaka, Japan; 5 Laboratory of Community Health and Nutrition, Department of Bioscience, Graduate School of Agriculture, Ehime University, Matsuyama, Ehime, Japan; 6 Department of Food Sciences and Nutrition, School of Food Sciences and Nutrition, Mukogawa Women’s University, Nishinomiya, Hyogo Japan; 7 Department of Endocrinology, First Affiliated Hospital of Kunming Medical University, Kunming, Yunnan China; 8 Department of Medicine, Kohnan Kakogawa Hospital, Kakogawa, Hyogo Japan

**Keywords:** Genetics, Medical research

## Abstract

Limited expandability of subcutaneous adipose tissue may be characteristics of first-degree relatives of type 2 diabetes. We tested the hypothesis that family history of type 2 diabetes (FHD) may be associated with reduced peripheral fat mass. Body composition and metabolic variables were compared between 18 and 111 Japanese female collegiate athletes, and between 55 and 148 nonathletes with positive (FHD +) and negative FHD (FHD-), respectively. We had multivariate logistic regression analyses for FHD + as dependent variable in a total population.BMI averaged < 21 kg/m^2^ and did not differ between FHD + and FHD- nonathletes. Despite comparable BMI, body fat percentage and serum leptin were lower in FHD + nonathletes. This was due to lower arm and gluteofemoral fat percentage (both *p* = 0.02) whereas the difference in trunk fat percentage was not significant (*p* = 0.08). These differences were not found between two groups of athletes. FHD + women had lower HDL cholesterol despite *lower* BMI in a total population. Fasting insulin, serum adiponectin and high-sensitivity C-reactive protein did not differ between FHD + and FHD- athletes or nonathletes. Multivariate logistic regression analyses revealed independent associations of FHD + with BMI (odds ratio, 0.869; 95% confidential interval, 0.768–0.984; *p* = 0.02) and HDL cholesterol (odds ratio, 0.977; 95% confidential interval, 0.957–0.997, *p* = 0.02). In **c**onclusion, FHD may be associated with reduced subcutaneous fat mass in young Japanese women, suggesting impaired adipose tissue expandability.

## Introduction

Excess dietary calories can be stored in subcutaneous adipose tissue (SAT), the largest and best storage site as fat. Excess lipids can be accumulated by an increase in the number of differentiated adipose cells (hyperplastic obesity), adipose cell enlargement (hypertrophic obesity) or both^1,2^. However, cell expansion is the major way of accommodating excess lipids since SAT is limited in its ability to recruit new cells in adults^3,4^. It is well-known that hypertrophic, rather than hyperplastic, obesity is closely associated with insulin resistance^1,2^. Hypertrophic obesity is also associated with reduced ability to recruit and differentiate precursor cells into adipose cells (adipogenesis) in SAT^5,6^. Studies have suggested that restricted adipogenesis may be associated with genetic predisposition for type 2 diabetes (first-degree relatives)^7–9^.

Insulin resistance is not only a central hallmark of the metabolic syndrome but also a consistent feature of lipodystrophy characterized by a partial or complete lack of SAT^10^. Genome-wide association studies focused on insulin resistance have linked common variants to genes implicated in adipose biology and suggested that subtle forms of lipodystrophy contribute to type 2 diabetes and cardiometabolic disease risk at a population level. For example, Scott et al.^11^ reported that just over half the loci associated with higher fasting insulin were associated with higher triglyceride (TG), lower high-density lipoprotein (HDL) cholesterol, and a lower BMI and/or a reduction in gluteofemoral fat mass as measured by dual X-ray absorptiometry (DXA) scanning. In a much larger study^12^, higher insulin-resistance genetic scores were associated with lower adipose mass in peripheral compartments by DXA. Interestingly, reduced expansion of gluteofemoral fat depot was also documented in response to weight gain^12^. We previously reported weight history since young adults in relation to carotid atherosclerosis in Japanese patients with type 2 diabetes^13^. More than half of patients (55%) reported positive family history of type 2 diabetes (FHD) and two-thirds of them were lean or normal weight at the time of the study with a mean BMI of 24.1 kg/m^2^, suggesting that FHD may be associated with the degree of adiposity in Japanese patients with type 2 diabetes^13^. To the best to our knowledge, studies on the association between fat mass and distribution and FHD are missing in Asian populations. Therefore, the purpose of the present study was to test the hypothesis that FHD may be associated with reduced peripheral fat mass. We studied Japanese women because men and women have different body fat distribution regulated by sex steroids^14^ and because Asian populations have a reduced ability to expand the SAT^5^.

## Methods

We cross-sectionally studied a total of 332 young Japanese women: 129 collegiate athletes and 203 female nonathletes with FHD data whose details were previously reported elsewhere^15^. As previously reported in details^16^, athletes were students of the Department of Health and Sports Sciences and were also members of the volleyball club, basketball club, or track club (middle-distance runners) of the University. They had been training regularly for 2 years or longer before the study, 5 h a day, and 6 days a week, and participated regularly in competitive events in their respective sport specialties. Nonathletes were students of the Department of Food Sciences and Nutrition of the University and were not engaged in any regular sport activity. Eighteen athletes and 55 nonathletes were considered FHD positive (FHD +) as they reported that a parent or a grandparent were on anti-diabetic drugs. Unfortunately, information was not available on the extent of family history (i.e., how many family members have the condition) and the nature of the family history (paternal or maternal). Among 332 women who provided FHD data, 331 provided height and weight at age 12 and 15 years either through maternal health check notes or child health notebook records (issued by each municipal office). Participants were students of the Mukogawa Women’s University and recruited as volunteers. Subjects with clinically diagnosed acute or chronic inflammatory diseases, endocrine, cardiovascular, hepatic, renal diseases, hormonal contraception, and unusual dietary habits were excluded. Nobody reported to receive any medications or have regular supplements. The study was approved by the Ethics Committees of the Mukogawa Women’s University (No. 07-28 on 19/02/2008) to be in accordance with the Helsinki declaration. All subjects gave written informed consent after the experimental procedure had been explained.

After a 12-h overnight fast at 8:30 A.M., height, weight and waist were measured. Blood was drawn to measure glucose, insulin, liver enzymes, serum and HDL cholesterol, TG, free fatty acid (FFA), adiponectin, leptin and high-sensitivity CRP (hsCRP)^17^. Homeostasis model assessment-insulin resistance (HOMA-IR) was calculated as the product of fasting insulin and glucose^18^. Insulin resistance in adipose tissue was evaluated by adipose tissue-insulin resistance index (AT-IR), which was calculated by the product of fasting insulin and FFA. We have recently shown that AT-IR may be a simple and useful surrogate index of adipose tissue insulin resistance even in Japanese women without diabetes and obesity^19^.

Fat mass, bone mass and lean mass for arms, lower-body, trunk and the total body were measured using whole-body DXA (Hologic QDR-2000, software version 7.20D, Waltham, MA) as previously reported^17^. Trunk fat included intra-abdominal and abdominal subcutaneous fat and hence android fat. Lower-body fat includes gluteal and leg fat and hence gluteofemoral (subcutaneous) fat or gynoid fat. DXA scans provided absolute and fat percentage for arms, lower-body, trunk and the total body. Fat percentage was used for the evaluation of fat mass. General fat accumulation was assessed by BMI and fat mass index (FMI), the latter of which was calculated by body fat mass in kg divided by height in meter squared. Abdominal/ central fat accumulation was assessed by waist and trunk fat percentage.

Data were presented as mean ± SD. First, we analyzed athletes and nonathletes separately because we have previously shown marked differences in body composition^16^. For example, 174 athletes vs. 311 nonathletes had higher lean mass in total body (42.9 ± 4.3 vs. 34.2 ± 3.1 kg, *p* < 0.001) and lower-body (15.2 ± 1.8 vs. 11.7 ± 1.3 kg, *p* < 0.001) and lower trunk fat (6.5 ± 2.1 vs. 7.0 ± 2.5 kg, *p* = 0.03) and serum leptin (6.4 ± 2.9 vs. 8.6 ± 3.9 ng/mL, *p* < 0.001)^16^. However, lower-body (leg) fat (5.5 ± 1.6 vs. 5.6 ± 1.5 kg, *p* = 0.83) and serum adiponectin did not differ^16^. Next, both groups were combined to analyze biochemical variables because of no or modest differences in serum lipids, lipoproteins and adiponectin between the two groups of women^16^. Due to deviation from normal distribution, HOMA-IR and hsCRP were logarithmically transformed for analyses. Differences between FHD + and FHD- were compared by t test. We did multivariate logistic regression analyses for FHD + as dependent variable in a total population of nonathletes and athletes. Independent variables included were age and variables that showed significant differences between FHD + and FHD−: BMI at age 20 years and HDL cholesterol. The sample size calculation was not conducted prior to the study. A two-tailed value of *p* < 0.05 was considered significant. Statistics were performed with SPSS system 21 (SPSS Inc, Chicago, IL).

## Results

When nonathletes and athletes were analyzed separately (Tables 1 and 2), BMI averaged < 21 kg/m^2^, waist < 75 cm, fasting glucose < 85 mg/dL, triglyceride < 65 mg/dL and insulin < 7 μU/mL and did not differ between FHD + and FHD−.

**Table 1:** Body composition of young Japanese women in the presence and absence of family history of type 2 diabetes (FHD).

	Nonathletes		Athletes	
	Positive FHD	Negative FHD	Positive FHD	Negative FHD
	n = 55	n = 148	n = 18	n = 111
Age (years)	20.5 ± 1.0	20.3 ± 1.2	19.7 ± 1.2	19.6 ± 1.2
Height (cm)	159.5 ± 5.5	158.7 ± 4.9	163.3 ± 6.4	165.1 ± 6.1
Weight (kg)	51.1 ± 8.6	51.7 ± 6.3	56.7 ± 6.5	59.1 ± 6.6
BMI (kg/m^2^)	20.0 ± 2.9	20.5 ± 2.1	21.2 ± 1.5	21.7 ± 2.0
Waist (cm) ^a^	72.6 ± 5.7	72.3 ± 6.2	72.5 ± 4.5	76.0 ± 4.7
FMI (kg/m^2^)	5.4 ± 2.0	5.9 ± 1.6	4.6 ± 1.3	5.0 ± 1.5
Trunk/leg fat ratio	1.3 ± 0.3	1.2 ± 0.2	1.2 ± 0.2	1.2 ± 0.2
Arm fat (kg)	1.1 ± 0.7	1.3 ± 0.6	0.9 ± 0.5	1.1 ± 0.5
Leg fat (kg)	5.3 ± 1.9	5.8 ± 1.4	5.1 ± 1.4	5.5 ± 1.4
Trunk fat (kg)	6.8 ± 3.0	7.2 ± 2.4	5.9 ± 1.8	6.5 ± 2.1
Total fat (kg)	13.8 ± 5.5	14.9 ± 4.2	12.4 ± 3.6	13.6 ± 3.8
**Percent arm fat (%)**	**23.3** ± **9.7**	26.7 ± 8.0*	17.3 ± 6.7	19.4 ± 6.8
**Percent leg fat (%)**	**29.3 ± 5.9**	31.4 ± 4.8*	24.3 ± 5.0	25.1 ± 4.6
Percent Trunk Fat (%)	27.7 ± 7.5	29.5 ± 6.2	22.0 ± 5.4	23.1 ± 5.6
**Percent total fat (%)**	**26.7 ± 6.5**	28.7 ± 5.2*	21.6 ± 4.9	22.7 ± 4.8
Arm lean mass (kg)	3.2 ± 0.5	3.1 ± 0.4	4.0 ± 0.5	4.2 ± 0.5
Leg lean mass (kg)	11.7 ± 1.4	11.6 ± 1.3	14.6 ± 1.7	15.2 ± 1.8
Trunk lean mass (kg)	16.1 ± 1.6	16.0 ± 1.5	19.7 ± 2.2	20.3 ± 2.1
Total lean mass (kg)	34.2 ± 3.5	33.9 ± 3.2	41.6 ± 4.4	43.1 ± 4.4
ASM (kg)	14.9 ± 1.9	14.7 ± 1.7	18.6 ± 2.2	19.4 ± 2.3
ASMI (kg/m2)	5.8 ± 0.6	5.8 ± 0.5	7.0 ± 0.4	7.1 ± 0.6

Mean ± SD.

^a^n = 48 and 64 in nonathletes and n = 9 and 32 in athletes with positive and negative family history, respectively.

ASM: appendicular skeletal muscle mass, ASMI: ASM index, BMI: body mass index, FMI: fat mass index.

Bold letters indicate *p  <  0.05.

**Table 2:** Biochemical characteristics of young Japanese women in the presence and absence of family history of type 2 diabetes (FHD).

	Nonathletes		Athletes	
	Positive FHD	Negative FHD	Positive FHD	Negative FHD
	n = 55	n = 148	n = 18	n = 111
**Leptin (ng/mL)**	**7.3 ± 3.8**	9.0 ± 4.0*	6.5 ± 2.3	6.5 ± 3.0
Adiponectin (mg/L)	12.0 ± 5.1	11.3 ± 4.0	11.3 ± 3.4	11.7 ± 4.5
hsCRP (μg/dL)	27 ± 44	29 ± 71	40 ± 116	27 ± 69
Fasting glucose (mg/dL)	83 ± 7	84 ± 7	88 ± 8	86 ± 7
Fasting insulin (μU/mL)	5.4 ± 3.1	6.2 ± 3.7	5.9 ± 2.5	6.3 ± 5.4
FFA (mEq/L)	0.59 ± 0.24	0.55 ± 0.21	0.43 ± 0.27	0.36 ± 0.16
HOMA-IR	1.1 ± 0.8	1.3 ± 0.9	1.2 ± 0.6	1.4 ± 1.3
AT-IR ^a^	3.3 ± 2.8	2.9 ± 1.9	2.5 ± 1.5	2.5 ± 1.6
Triglyceride (mg/dL)	61 ± 57	57 ± 23	52 ± 18	56 ± 23
HDL cholesterol (mg/dL)	71 ± 12	74 ± 13	76 ± 12	79 ± 14

Mean ± SD.

^a^n = 48 and 64 in nonathletes and n = 9 and 32 in athletes with positive and negative family history, respectively.

hsCRP: high-sensitivity C-reactive protein, FFA: free fatty acid, HOMA-IR: homeostasis model assessment-insulin resistance, AT-IR: adipose tissue-insulin resistance index.

Bold letters indicate *p  =  0.007.

**Figure 1:**
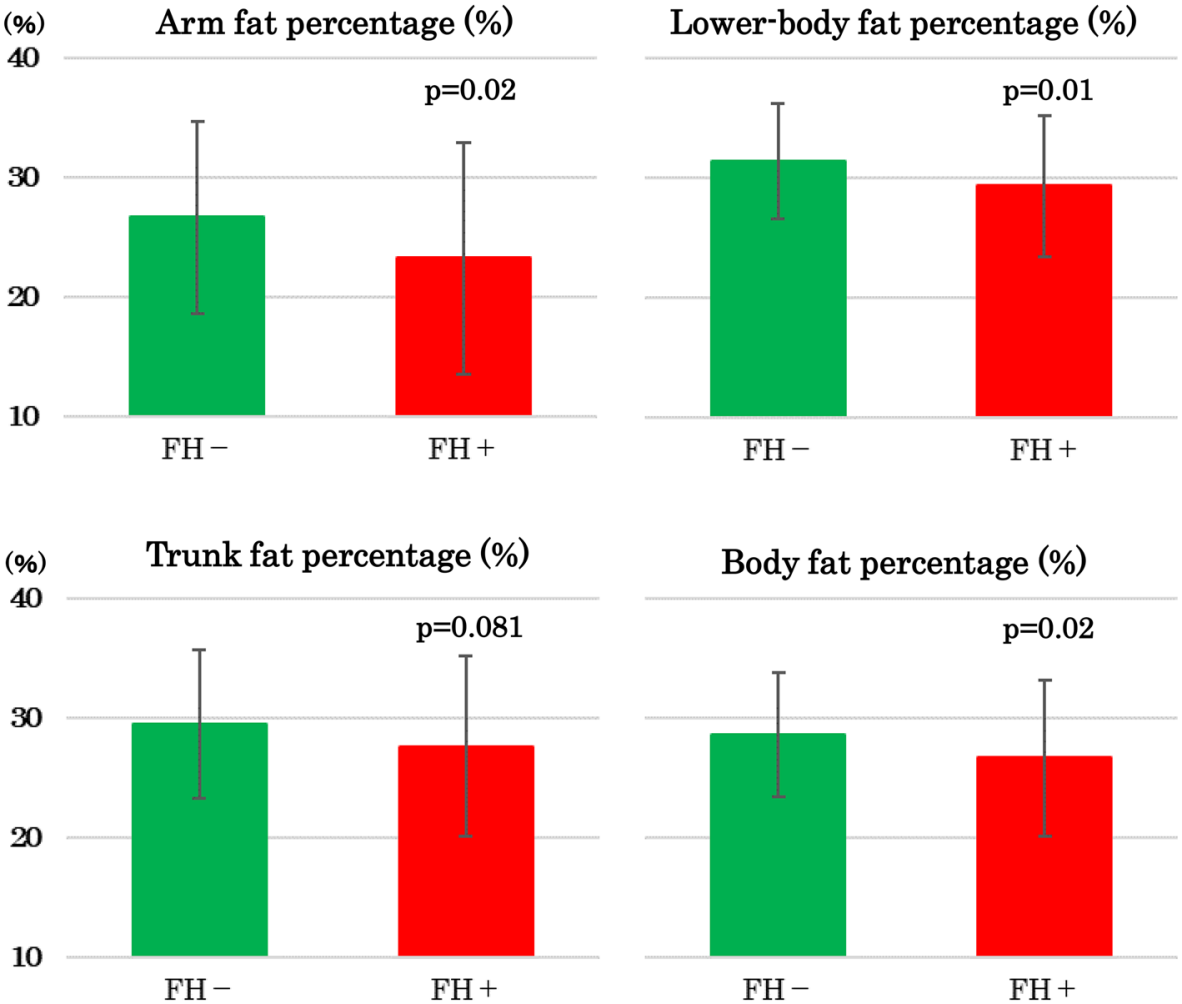
Fat percentage in arms, lower-body, trunk, and entire body in female nonathletes students in the presence (FH + , red columns, n = 55) and absence (FH-, green columns, n = 148) of family history of type 2 diabetes. Lower-body includes gluteal and leg (gluteofemoral) region. Note that differences in arms and lower-body were significant while that in trunk was not.

In spite of comparable BMI, body fat percentage was lower, and FMI was tended to be lower in nonathletes with FHD + compared with nonathletes without FHD (Fig. 1 and Table 1). Lower body fat percentage was due to lower fat percentage in arms, legs and gluteal region whereas the difference in trunk fat percentage was not significant (Fig. 1). There was no difference in lean mass in any regions studied.

Female nonathletes with FHD had lower serum leptin whereas serum adiponectin and hsCRP did not differ (Table 2). There was also no difference in HDL cholesterol, TG, HOMA-IR and AT-IR. FHD was not associated with body fat percentage, trunk fat percentage and leptin in multivariate logistic regression analyses in nonathletes (data not shown).

In female athletes (Tables 1 and 2), there was no significant difference in body composition and biochemical variables studied between women with positive and negative FHD.

When athletes and nonathletes were combined (Table 3), height and weight at age 12 and 15 years did not differ between FHD + and FHD- women. However, although height at age 20 years did not differ, weight and hence BMI were lower in FHD + women. i.e., weight gain between 15 and 20 years of age was less in FHD + compared to FHD- women. Lower-body fat mass (Table 3) and serum leptin (Table 4) tended to be lower in FHD + women although there was no difference in absolute body fat, height-adjusted and weight-adjusted body fat (FMI and body fat percentage, respectively). Lean mass in the four regions, ASM, ASMI and a percentage of athletes were all lower in FHD + compared to FHD- women.

**Table 3:** Anthropometric characteristics of young Japanese female athletes and non-athletes in the presence and absence of family history of type 2 diabetes (FHD).

	Positive FHD	Negative FHD	*p* values
	n = 73	n = 259	
Age (years)	20.3 ± 1.1	20.0 ± 1.2	0.079
Height (cm) at age 12	152.4 ± 5.9	153.1 ± 7.5	0.456
at age 15	158.7 ± 5.8	159.7 ± 6.4	0.259
at age 20	160.4 ± 6.0	161.4 ± 6.3	0.243
Weight (kg) at age 12	44.4 ± 7.6	45.0 ± 7.5	0.538
at age 15	51.0 ± 8.0	51.8 ± 7.2	0.422
at age 20	52.5 ± 8.5	54.9 ± 7.4	0.021
BMI (kg/m^2^) at age 12	19.0 ± 2.4	19.1 ± 2.2	0.811
at age 15	20.2 ± 2.6	20.3 ± 2.1	0.747
at age 20	20.3 ± 2.6	21.0 ± 2.1	0.023
Waist (cm) ^a^	72.6 ± 5.5	73.6 ± 6.0	0.323
FMI (kg/m^2^)	5.2 ± 1.9	5.5 ± 1.6	0.161
Trunk/lower-body fat ratio	1.2 ± 0.2	1.2 ± 0.2	0.689
Arm fat (kg)	1.1 ± 0.7	1.2 ± 0.6	0.115
lower-body fat (kg)	5.3 ± 1.8	5.7 ± 1.4	0.063
Trunk fat (kg)	6.5 ± 2.7	6.9 ± 2.3	0.261
Total body fat (kg)	13.5 ± 5.1	14.3 ± 4.1	0.131
Arm fat percentage (%)	21.8 ± 9.4	23.6 ± 8.3	0.123
lower-body fat percentage (%) =	28.1 ± 6.1	28.7 ± 5.7	0.422
Trunk fat percentage (%)	26.3 ± 7.4	26.8 ± 6.7	0.599
Body fat percentage (%)	25.4 ± 6.5	26.1 ± 5.8	0.395
Arm lean mass (kg)	3.4 ± 0.6	3.6 ± 0.7	0.085
lower-body lean mass (kg)	12.4 ± 2.0	13.2 ± 2.4	0.006
Trunk lean mass (kg)	17.0 ± 2.4	17.8 ± 2.8	0.011
Total lean mass (kg)	36.1 ± 4.9	37.9 ± 5.9	0.009
ASM (kg)	15.8 ± 2.5	16.7 ± 3.0	0.009
ASMI (kg/m^2^)	6.1 ± 0.7	6.4 ± 0.8	0.014
Athletes (n, %)	18, 24.7	111,42.9	0.003

Mean ± SD or n, %.

^a^n = 57 and 96 of positive and negative family history, respectively. Abbreviations are the same as in Table 1.

**Table 4:** Biochemical characteristics of young Japanese female athletes and non-athletes in the presence and absence of family history of type 2 diabetes (FHD).

	Positive FHD	Negative FHD	*p* values
	n = 73	n = 259	
Leptin (ng/mL)	7.1 ± 3.5	7.9 ± 3.8	0.094
Adiponectin (mg/L)	11.8 ± 4.7	11.5 ± 4.2	0.523
hsCRP (μg/dL)	30 ± 68	28 ± 70	0.811
Fasting glucose (mg/dL)	84 ± 7	85 ± 7	0.497
Fasting insulin (μU/mL)	5.5 ± 3.0	6.2 ± 4.5	0.216
FFA (mEq/L)^a^	0.56 ± 0.25	0.49 ± 0.21	0.064
HOMA-IR	1.1 ± 0.7	1.3 ± 1.0	0.182
AT-IR^a^	3.2 ± 2.7	2.8 ± 1.8	0.259
Triglyceride (mg/dL)	59 ± 50	57 ± 23	0.691
HDL cholesterol (mg/dL)	72 ± 12	76 ± 14	0.025
ALT (U/L)	12 ± 4	14 ± 8	0.082
GGT (U/L)	13 ± 4	14 ± 5	0.139

Mean ± SD.

^a^n = 57 and 96 of positive and negative family history, respectively.

ALT: alanine aminotransferase, GGT: γ-glutamyl transferase, other abbreviations are the same as in Table 2.

As shown in Table 4, HDL cholesterol was lower despite *lower* BMI in women with FHD. There was no difference in serum adiponectin, inflammatory markers, triglycerides and liver enzymes.

In multivariate logistic regression analyses in a total population of nonathletes and athletes for FHD + as a dependent variable, BMI at age 20 years (odds ratio, 0.869; 95% confidential interval, 0.768–0.984; *p* = 0.02) and HDL cholesterol (odds ratio, 0.977; 95% confidential interval, 0.957–0.997, *p* = 0.02) were associated with FHD + .

## Discussion

The present study has demonstrated that young normal weight Japanese female nonathletes with FHD had reduced body fat percentage and serum leptin, a sensitive marker of body fat mass^20^, despite comparable BMI. Reduced body fat percentage was due to reduced fat mass in limb and gluteal region. However, body fat percentage and serum leptin did not differ between FHD + and FHD- athletes. When athletes and non-athletes were combined, women with FHD had lower HDL cholesterol despite *lower* BMI. Although low HDL cholesterol may be due in part to low percentage in athletes in women with FHD, low HDL cholesterol and low BMI were associated with FHD + in multiple logistic regression analyses.

Lipodystrophies, characterized by a partial or complete lack of white adipose tissue, almost all cause insulin resistance, nonalcoholic fatty liver disease, and dyslipidemia (characterized by high TG and low HDL cholesterol)^10^. GWAS focused on insulin resistance suggested that subtle forms of lipodystrophy contribute to cardiometabolic disease risk at a population level^11,12^. For example, Lotta et al.^12^ reported that higher insulin-resistance genetic scores were associated with lower percent body fat, BMI, leg fat mass by DXA. As summarized by Mann and Savage^10^, the genetic score was enriched in patients with familial partial lipodystrophy type 1 (FPLD1), which is an extreme form of apple-shaped fat distribution, implying that these common alleles contribute to both common insulin resistance and a specific form of partial lipodystrophy known as FPLD1. This is also consistent with observations that there are a large number of patients with clinical features of FPLD with no known genetic diagnosis^10^. Young Japanese women with FHD in the present study had a subtle lipodystrophy-like phenotype: reduced fat in limb and gluteal region and relatively preserved trunk fat. This subtle lipodystrophy-like body composition is similar to those in patients with FPLD3 characterized by the preservation of abdominal fat with loss of limb and gluteal fat depots^21^. Lower HDL cholesterol despite lower BMI in women with FHD, in analyses where athletes and non-athletes were combined, may be related to lipid dysmetabolism in subtle lipodystrophy-like body composition. Studies have suggested that restricted adipogenesis may be associated with genetic predisposition for type 2 diabetes (first-degree relatives)^7–9^. Therefore, we assume that limited expandability of adipose tissue in Japanese women with FHD is genetically determined.

The association between FHD and HDL cholesterol, while statistically significant, was very subtle in terms of effects on metabolic traits. Moreover, HDL cholesterol concentrations in women with FHD were relatively high (72 mg/dL). Therefore, our results do not suggest that a lipodystrophy-like phenotype is the primary mechanism of insulin resistance and its link to metabolic abnormalities. Instead, although we did not measure adipogenesis, but only quantified fat mass, our results suggest that a reduced ability to expand gluteofemoral fat may be one mechanism that links FHD with low HDL cholesterol despite low BMI in young Japanese women as studies showed that lower gluteofemoral fat mass was associated with higher risk of diabetes and cardiovascular disease and that this increased risk was independent of increased trunk fat mass^22,23^.

Waist averaged 73 cm and serum ALT 12 U/L in women with FHD, suggesting minimum abdominal and hepatic lipid accumulation, respectively. Further, HOMA-IR averaged 1.1, suggesting that they were not in insulin resistant states although they were less insulin sensitive than women without FHD. The hallmark of dyslipidemia in dysfunctional white adipocytes in obesity is low HDL cholesterol levels^24^ whereas high triglyceride concentrations were the hallmark of dyslipidemia in excess abdominal and hepatic lipid accumulation. The overproduction of FFA and very low-density lipoprotein TG is regarded as one of the reasons responsible for low HDL cholesterol level^25^. Taken together, these observations may be related to present findings that lower gluteofemoral fat mass was not associated with any indices of insulin resistance other than lower HDL cholesterol in young Japanese women with FHD.

We previously reported weight history since young adults in relation to carotid atherosclerosis in Japanese patients with type 2 diabetes, in whom 67% were lean or normal weight at the time of the study and 55% reported FHD positive^13^. We investigated retrospectively weight history in relation to family history. Among patients whose current BMI < 25 kg/m^2^, 55% (37% of total) reported that their BMI never exceeded over 25 kg/m^2^, in whom 53% (20% of total) reported FHD positive i.e., one in five patients studied were not only FHD positive but also their BMI never exceeded over 25 kg/m^2^.

As described earlier and reported previously^16^, athletic compared with nonathletic female university students had lower body fat percentage, lower serum leptin and higher lean mass. These findings may be associated with no difference in body fat percentage and serum leptin between FHD + and FHD- athletes in the present study. Lower lean mass in the four regions, ASM and ASMI in FHD + compared to FHD- women in a total population (Table 3) is due to a lower percentage of athletes in FHD + women.

A homogeneous study population with scarce confounding factors is a strength of the present study as previously reported^17^. For example, more than 90% of grade 1 students are 18 years old. This approach may decrease confounding factors, including smoking, alcohol, educational and socioeconomic status whereas it may increase genetic load^17^. Another strength is accurate and reliable measures of DXA-derived body composition. However, DXA does not allow separate quantification of intra-abdominal fat and abdominal subcutaneous fat in the trunk. There are also several limitations including a lack of detailed information about FHD, the cross-sectional design and a single measurement of biochemical variables. The recruitment procedure may also have some potential impact on the results. Women who pay more attention to health may be more likely to participate as the participation was voluntary. We used crude measures of insulin resistance, which may be less accurate. Statistical power was not calculated. As we studied young Japanese women only, results may not be generalized to other gender, age populations, races or ethnicities.

In conclusion, young Japanese women with FHD had lower HDL cholesterol, a marker of insulin resistance, despite *lower* BMI. Their lower body fat percentage was a consequence of lower subcutaneous (limb and gluteal) fat mass, a subtle lipodystrophy-like phenotype. Taken together, FHD may be associated with reduced subcutaneous fat mass in young Japanese women, suggesting impaired adipose tissue expandability. Therefore, young Japanese women with FHD should pay attention to future weight control, during pregnancy in particular, because it has been reported that a weight gain of 5.0 to 7.9 kg in women increased the risk of developing diabetes by 1.9 times independently of FHD^26^ and because FHD is a risk factor of gestational diabetes^27^.

## Data Availability

The datasets used and/or analysed during the current study available from the corresponding author on reasonable request.
